# Unlawfully denying patients general practice access should be considered a ‘never event’

**DOI:** 10.3399/BJGP.2026.0328

**Published:** 2026-09-01

**Authors:** Fred Barker, Claire Rees, Joy Anderson, John Ford

**Affiliations:** 1 Wolfson Institute of Population Health, Queen Mary University of London, London, UK

While access to general practice is a right for all patients based in England, large numbers of eligible patients are being regularly refused registration. We argue that classifying these cases as ‘never events’ would mobilise changes at practice level, significantly improving healthcare access for all affected patients.

## What are never events?

Never events are defined as *‘Serious Incidents that are wholly preventable because guidance or safety recommendations that provide strong systemic protective barriers are available at a national level’*.^1^ The purpose of defining these incidents in such a way is to lobby healthcare organisations to act wherever possible to stop them taking place.

England’s Never Events Policy and Framework lists specific criteria describing a never event; some are listed below:^1^

- there is evidence that the event has happened in the past;
- events are wholly preventable assuming adequate safeguards are in place;
- the event has the potential to cause serious patient harm or death; and
- there is existing national guidance aiming to prevent events occurring.

In cases where never events occur, a full investigation becomes mandatory in accordance with the Patient Safety Incident Response Framework^2^ (which recently replaced the Serious Incident Framework^1^). It also permits oversight by the Care Quality Commission (CQC), providing an additional layer of accountability to support improvements.

*“NHS policy states that all people living in England are entitled to register with a local GP practice, and that neither a home address nor an ID is required to be registered.”*

We note that NHS England proposed new changes to never events policy in September 2025, such as removing the assertion that never events are necessarily always ‘wholly preventable’.^3^ We believe our argument below remains valid in spite of all proposed changes.

## Evidence of unlawful GP registration refusals

NHS policy states that all people living in England are entitled to register with a local GP practice, and that neither a home address nor an ID is required to be registered.^4^ Some of the most vulnerable patient groups in England are also the most likely to struggle to provide these forms of documentation, such as people experiencing homelessness or seeking asylum, victims of human trafficking, and nomadic communities. However, a large quantity of evidence exists showing that, in spite of national guidance, patients lacking these forms of documentation are being frequently refused registration.

Friends, Families and Travellers (FFT) carried out two ‘mystery shopper’ assessments of practices in 2019 and 2021 to investigate this issue,^5^ where attempts were made to register as a theoretical patient without an address or ID. Out of the 150 GP practices contacted across the two assessments, 98 (65%) refused registration. Importantly, the report also found that these access issues were not identified by any CQC evaluations, who rated practices refusing registration as ‘good’ or ‘outstanding’ for the domain relating to supporting *‘people whose circumstances may make them vulnerable’*.^5^

Similar investigations have been carried out elsewhere, finding GP registration was refused in most cases.^6,7^ A 2019 study interviewed people who were homeless about their experiences of primary care access, finding that some believed there was ‘no way’ to be able to register with a GP in their circumstances.^8^ Cases were reported where a patient’s friends and relatives had to fraudulently address utility bills in their name to allow them to be able to register with a practice. Patients’ sensitive medical information being sent to incorrect addresses as a direct result of this is a serious confidentiality concern.

Interventions to address this to date have not been able to resolve the issue. The charity Doctors of the World (DOTW) audited its real-world attempts to assist GP registrations of migrant patients. The charity informed receptionists of the patients’ circumstances, offering to provide proof of address letters from DOTW, and signposted to national guidelines regarding GP registration. Despite this, 19% of attempts to register patients were still refused.^9^ Other local interventions have not been able to meaningfully improve access. A mystery shopper exercise in Waltham Forest found that proof of address was required for 73% of practices.^10^ Following this result, borough-wide education sessions and other interventions were implemented. However, a replication of the investigation the following year found that proof of address was required by receptionists more frequently — 87% of attempts.^11^

*“Denying people their legal right to general practice is a common, widespread example of systemic discrimination against vulnerable patient groups. This must be addressed urgently.”*

Much of the evidence listed above is taken from grey literature, particularly the audits of GP registration attempts; while findings are consistent across settings and organisations, peer-reviewed research would help strengthen confidence in the scale of the problem.

## Factors driving registration refusals

Carlson and Pepper’s study explored the reasons behind practices’ refusals of GP registration attempts.^12^ Some staff raised safety concerns when not able to confirm a patient’s identity. Others felt that these groups of patients would be at high risk of rejection by the NHS’s central approval system, increasing administrative workload for staff. Other alarming factors were at play, including beliefs that certain (eligible) patients should not be entitled to NHS primary health care or were ‘burdensome’. Interviewees often felt that the guidance provided was ambiguous, leading to misinterpretation by receptionists. In accordance with FFT’s findings, the study found that practices were not held accountable for refusing to register eligible patients — not a single member of interviewed practice staff had experienced negative feedback regarding their approaches.^12^ The necessity of robust accountability mechanisms in reducing health inequalities has been emphasised in NHS literature,^13^ as failing to develop these frameworks weakens incentives for improvement.

## Defining these circumstances as never events could resolve this issue

Never events were historically conceived to prevent procedural safety errors, though this need not limit their application. The healthcare access failures we describe meet all criteria required for never events and could therefore be described as such. We believe rapid improvements could be seen from this small change in policy alone.

English general practice is agile and adapts quickly to new policy, in part because of its small business-style partnership model. Changes required at practice level would be relatively small and would allow full autonomy in how these are implemented, minimising bureaucracy and unnecessary micro-management. At the same time, practices that fail to take appropriate action can be identified and held accountable. We acknowledge that this policy may lead to increased stress and administrative burden for practices, but additional workload would fall primarily on practices that continue to unlawfully refuse registration. Ultimately, we believe this process would be a constructive and worthwhile means of sustainably improving healthcare access.

Patients can already report unlawful registration refusals via the national Patient and Public Reporting eForm,^14^ though such reports are not currently investigated individually. We recommend that this channel is developed further to support investigation of never events of this kind, with practices identified through reports triggering the Patient Safety Incident Response Framework and CQC oversight.

Guidance currently suggests that requesting personal documentation is important during the registration process, despite asserting elsewhere that patients without it should not be refused.^4^ We agree with participants from Carlson and Pepper’s study that this is too ambiguous and could lead to misinterpretation. We therefore additionally recommend removing any encouragement to check IDs or home addresses at point of registration. Making this guidance change without never events policy may improve access to some extent, but we believe it is unlikely to be sufficient to overcome all barriers to access that have been identified.

## Conclusion

Denying people their legal right to general practice is a common, widespread example of systemic discrimination against vulnerable patient groups. This must be addressed urgently.

Classifying the incidents we have described as never events will not only encourage relevant practices to act but will also ensure that those who do not support equitable healthcare access can be held accountable. This would have significant positive impacts on health and wellbeing for all affected groups, constituting a much-needed shift towards a kinder and more equitable NHS.
